# Supplementary material for: Intrauterine growth and the tangential expansion of the human cerebral cortex in times of food scarcity and abundance
Source: Nat Commun. 2024 Feb 13;15:1205. doi: 10.1038/s41467-024-45409-6 (PMC10864407; doi:10.1038/s41467-024-45409-6)
Supplement: Supplementary file 1 — Supplementary Information [file 41467_2024_45409_MOESM1_ESM.pdf]

Supplementary Materials for

Intrauterine growth and the tangential expansion of the human cerebral cortex in times of food scarcity and abundance

Daniel E. Vosberg, Ph.D.[1,2], Igor Jurisica, Ph.D./Dr.Sc.[6,7,8], Zdenka Pausova, M.D.[3, 9,11], *Tomáš Paus, M.D./Ph.D.[1,2,10,11,12]

Corresponding author: tpausresearch@gmail.com

The PDF file includes:

Figs. S1 to S15

Other Supplementary Materials for this manuscript include the following:

Supplementary Data 1 to 2 (separate file: “supplementary_data.xlsx”)
Supplementary Software 1 (separate file: “map_mQTLs_famine_score_2023feb15.txt”)

Supplementary Figures

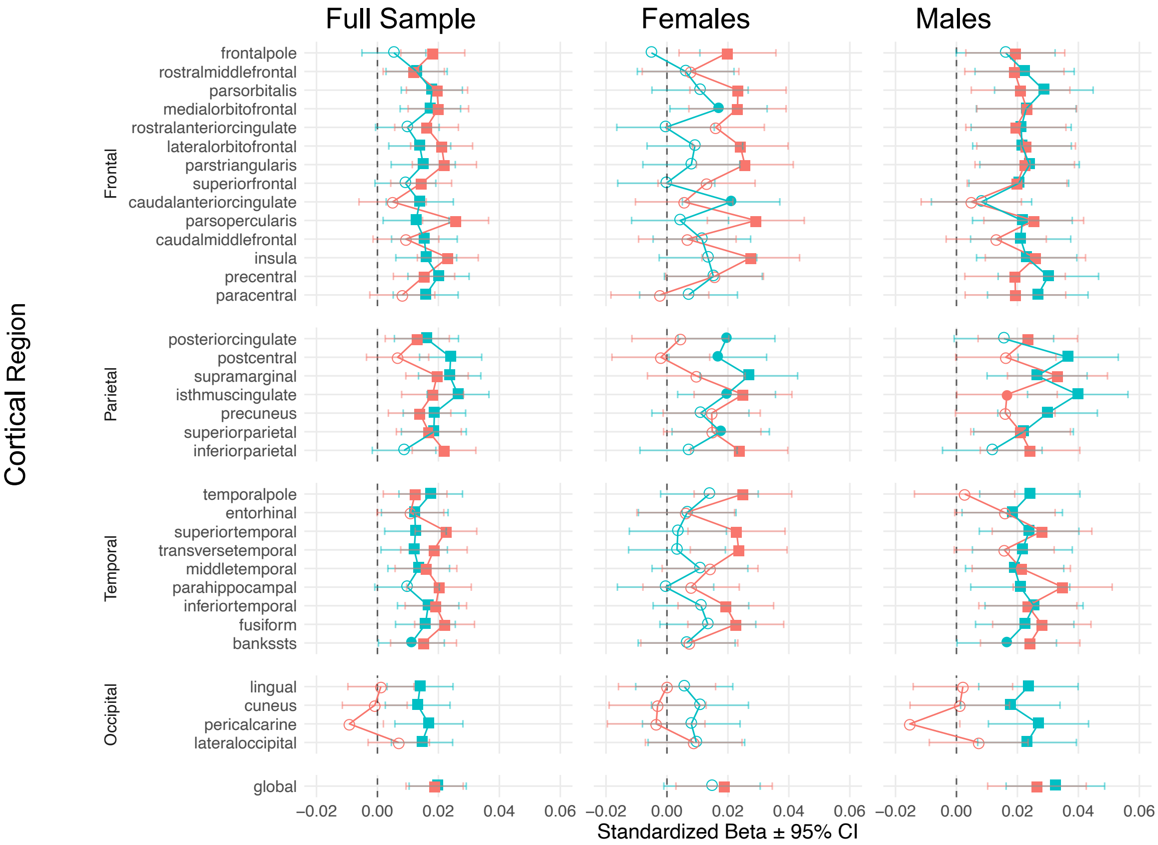

Fig. S1. Associations between Polygenic Scores (PGSs) for birthweight (back), fetal (red) and maternal (cyan) variants and cortical surface area, stratified by sex (total n = 29,047, male n = 14,142; female n = 14,905). The significance levels are indicated by filled squares ($p_{FDR} < 0.05$), filled circles ($p < 0.05$), and open circles ($p > 0.05$).

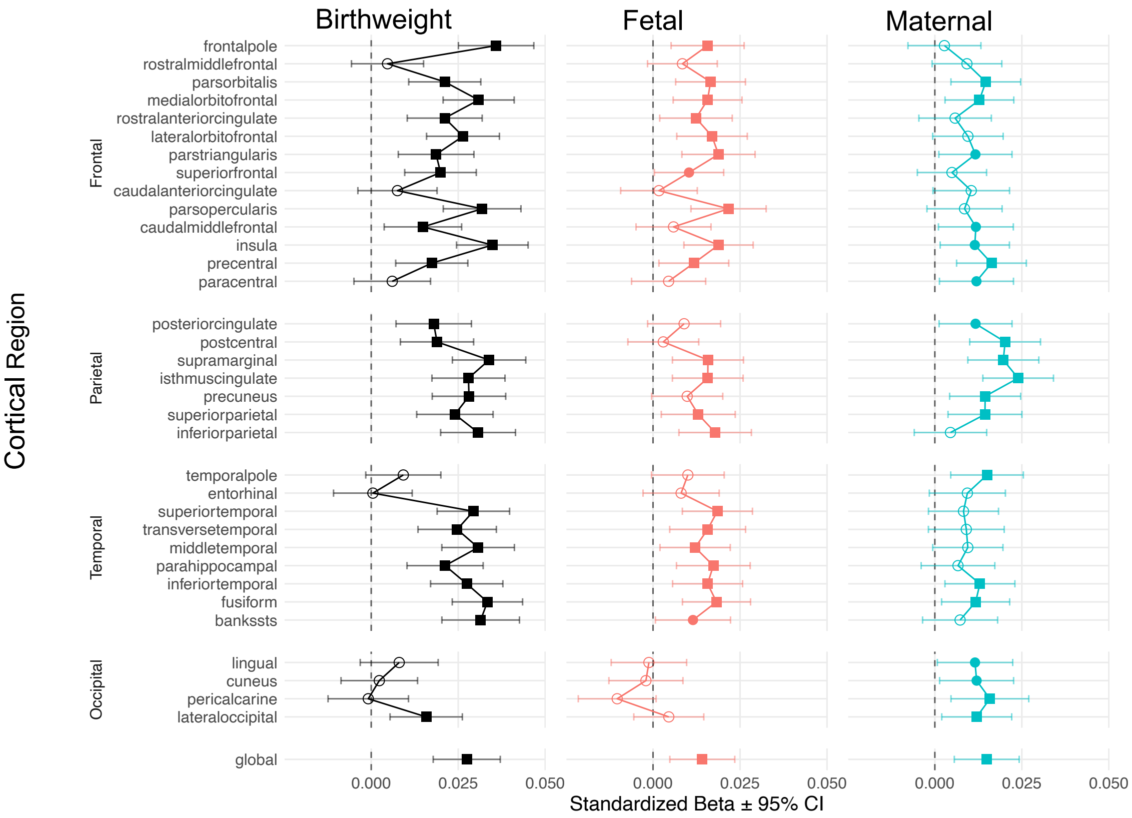

Fig. S2. Associations between Polygenic Scores (PGSs) for birthweight (back), fetal (red) and maternal (cyan) variants and cortical surface area, stratified by sex and adjusted for height PGS (total n = 29,047, male n = 14,142; female n = 14,905). The significance levels are indicated by filled squares ($p_{FDR} < 0.05$), filled circles ($p < 0.05$), and open circles ($p > 0.05$). Additionally, the correlations between the height and birthweight PGSs were significant but accounted for very little of the variance among females (fetal: $r^2 = 0.003$, p = 2.89e-10; maternal: $r^2 = 0.002$, p = 1.32e-09) and males (fetal: $r^2 = 0.002$, p = 2.00e-07; maternal: $r^2 = 0.003$, p = 4.58e-11).

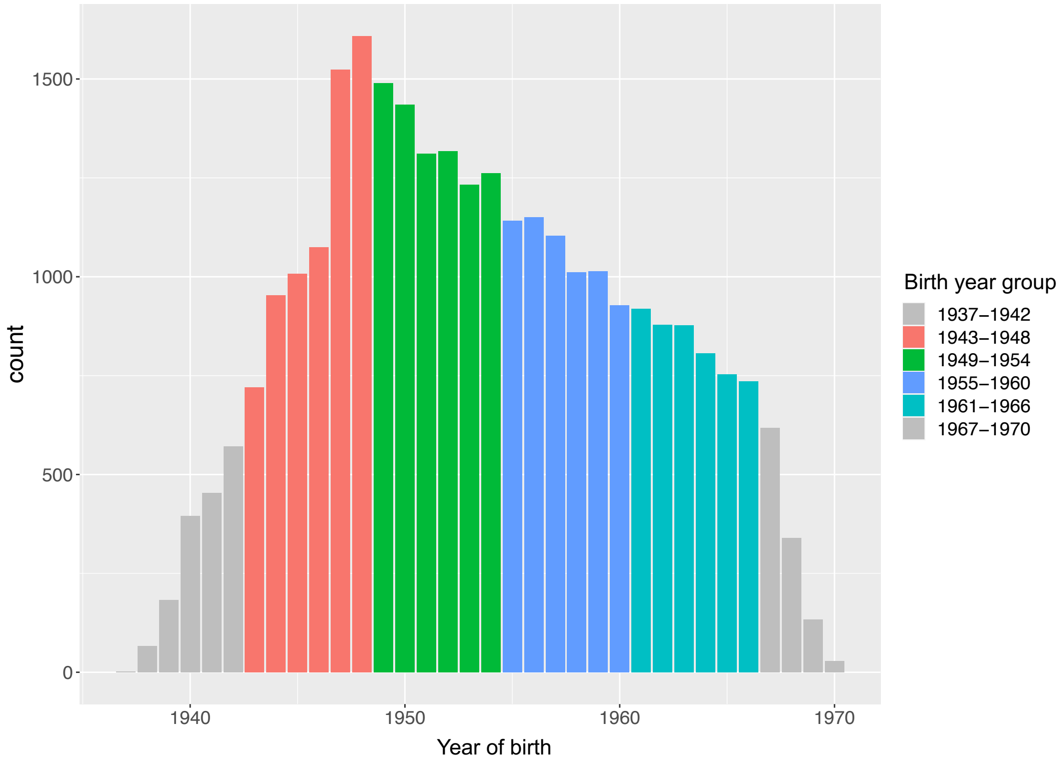

Fig. S3. The distribution of year of birth in the UK Biobank, indicating the number of participants by birth year periods: 1937-1942 (n = 1670), 1943-1948 (n = 6888), 1949-1954 (n = 8048), 1955-1960 (n = 6349), 1961-1966 (n = 4971), and 1967-1970 (n = 1121), with the lowest sample sizes in the oldest (1937-1942) and youngest (1967-1970) birth year groups.

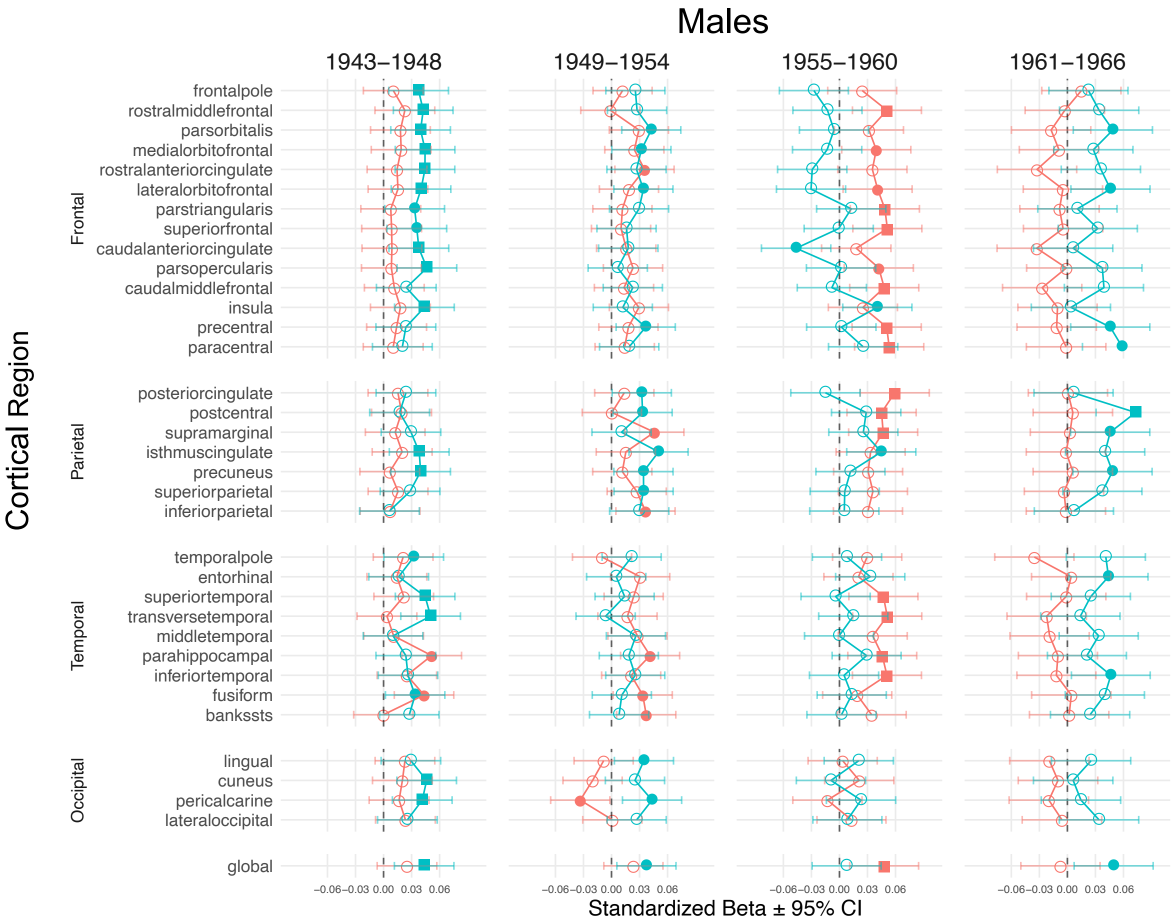

Fig. S4A. Associations between PGSs for fetal (red) and maternal (cyan) effects on birthweight and phenotypic cortical SA in males, segmented by year of birth period. The analyses were conducted in the following birth year periods: 1943-1948 (n = 3771), 1949-1954 (n = 3837), 1955-1960 (n = 2821), and 1961-1966 (n = 2154). The significance levels are indicated by filled squares ($p_{FDR} < 0.05$), filled circles ($p < 0.05$), and open circles ($p > 0.05$).

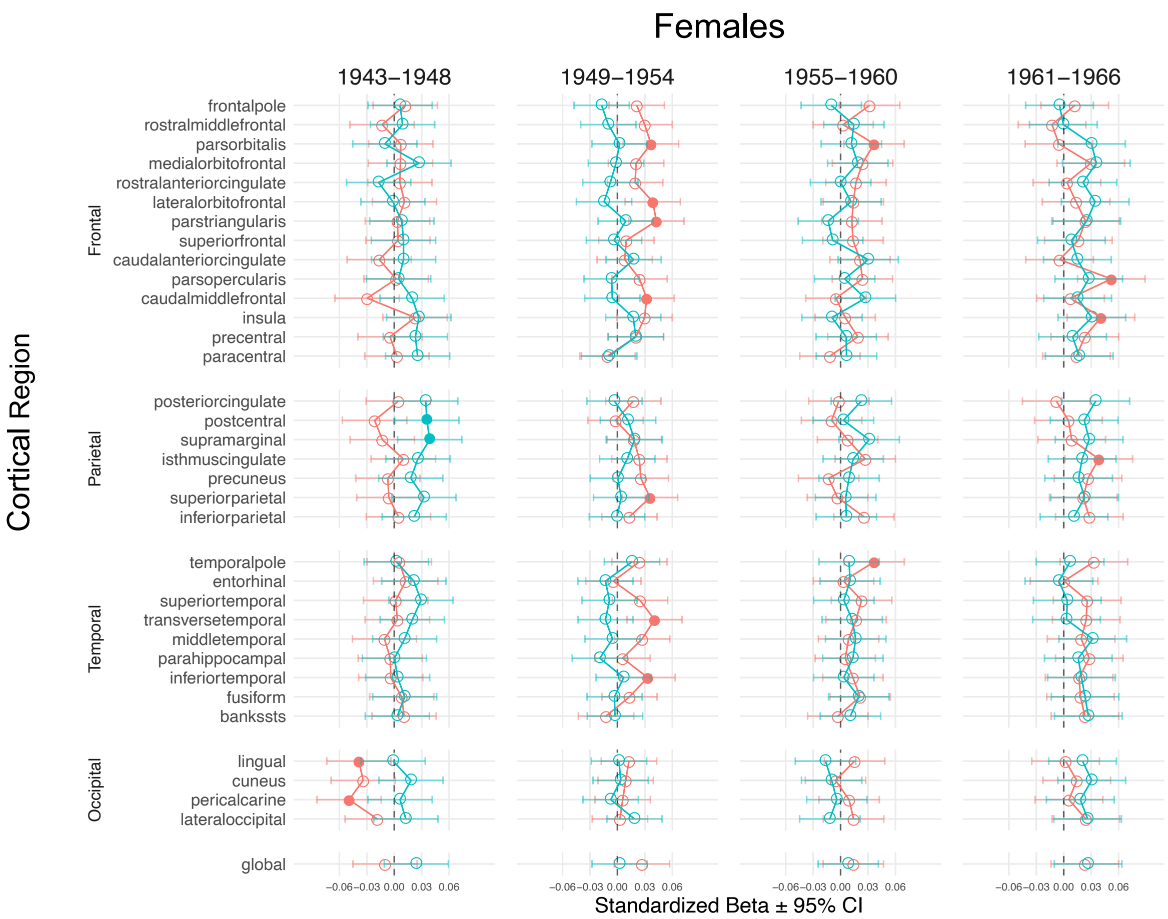

Fig. S4B. Associations between PGSs for fetal (red) and maternal (cyan) effects on birthweight and phenotypic cortical SA in females, segmented by year of birth period. The analyses were conducted in the following birth year periods: 1943-1948 (n = 3117), 1949-1954 (n = 4211), 1955-1960 (n = 3528), and 1961-1966 (n = 2817). The significance levels are indicated by filled squares ($p_{FDR} < 0.05$), filled circles ($p < 0.05$), and open circles ($p > 0.05$).

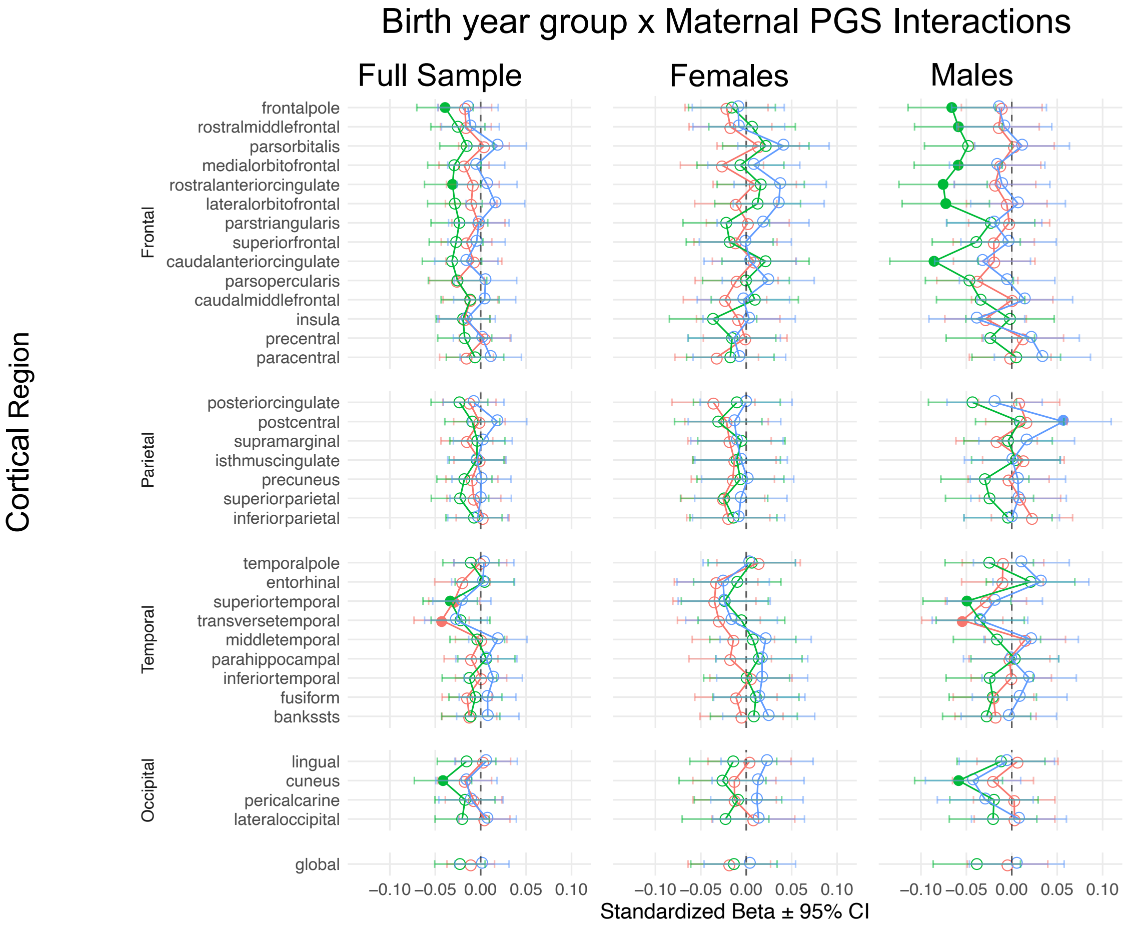

Fig. S5A. Assessment of maternal PGS *x* birth year group interactions for the combined sample, females, and males, in the UK Biobank (total n = 27,377, male n = 13,100; female n = 14,277). The three interaction effect contrasts compared the reference group (1943-1948) to each of the three other birth year groups (1949-1954 [red], 1955-1960 [green], 1961-1966 [blue]). The significance levels are indicated by filled squares ($p_{FDR} < 0.05$), filled circles ($p < 0.05$), and open circles ($p > 0.05$). Here, the FDR correction was applied for 105 p-values (35 regions x 3 interaction estimates), for the full sample, males, and females.

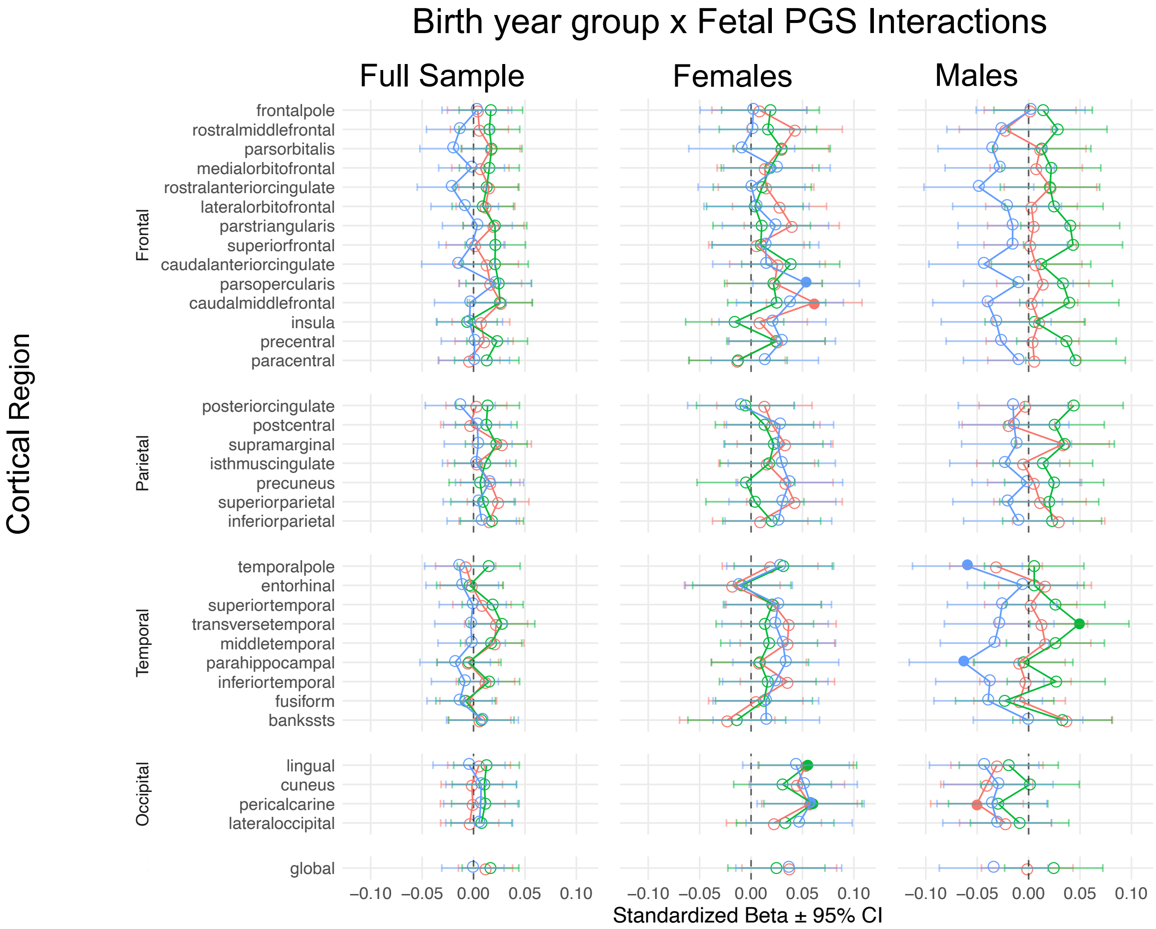

Fig. S5B. Assessment of fetal PGS *x* birth year group interactions for the combined sample, females, and males, in the UK Biobank (total n = 27,377, male n = 13,100; female n = 14,277). The three interaction effect contrasts compared the reference group (1943-1948) to each of the three other birth year groups (1949-1954 [red], 1955-1960 [green], 1961-1966 [blue]). The significance levels are indicated by filled squares ($p_{FDR} < 0.05$), filled circles ($p < 0.05$), and open circles ($p > 0.05$). Here, the FDR correction was applied for 105 p-values (35 regions x 3 interaction estimates), for the full sample, males, and females.

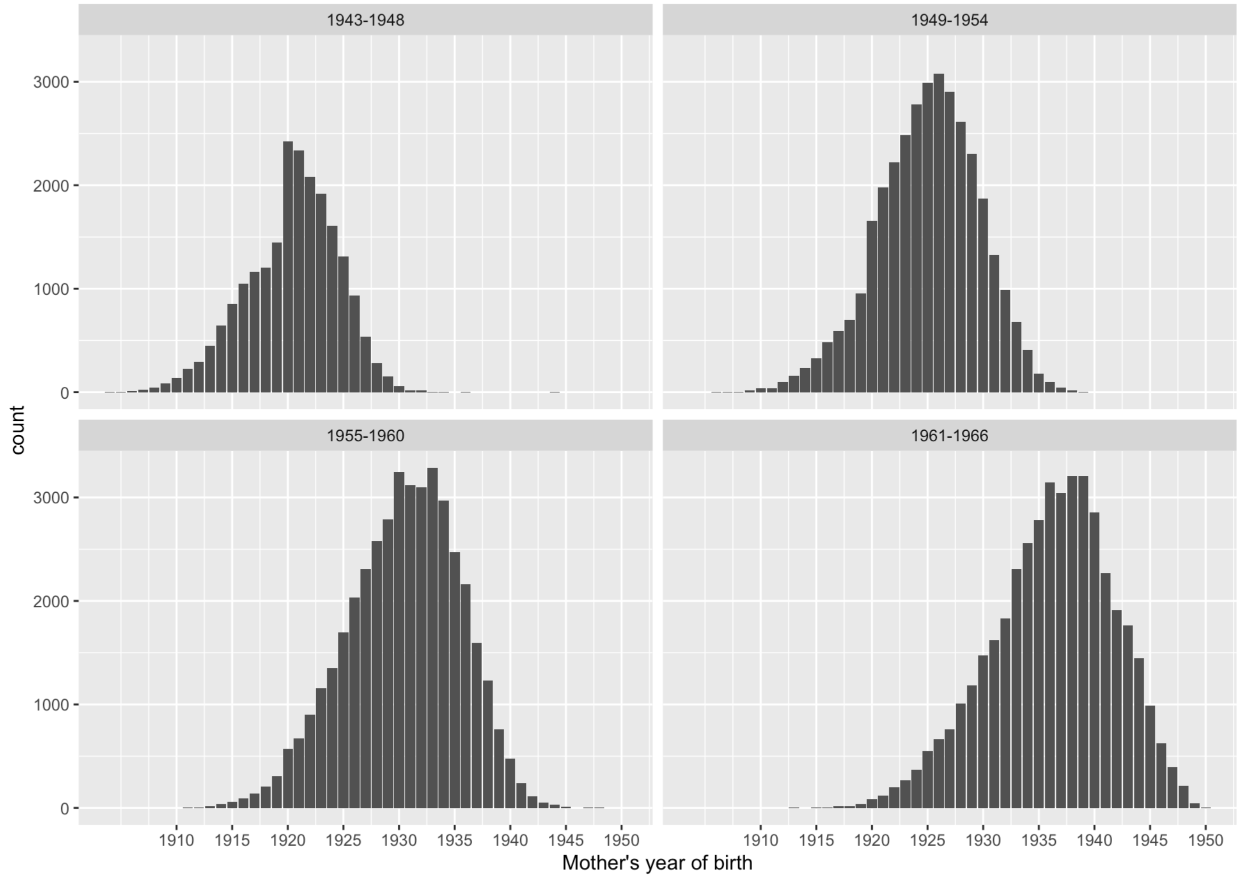

Fig. S6. Distributions for mothers' year of birth, for the four cohorts.

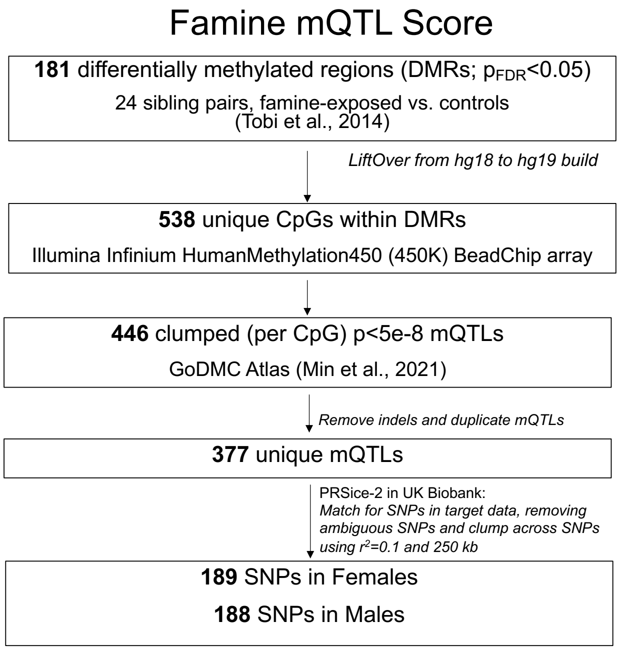

Fig. S7A. Identification of methylation Quantitative Trait Loci. First, we extracted the positions of the 181 differentially methylated regions (DMRs; $p_{FDR} < 0.05$) contrasting 24 famine-exposed cases and their sibling controls [28], using LiftOver from genome build hg18 to hg19. Within these 181 DMRs, we identified 538 unique CpGs contained in the Illumina Infinium HumanMethylation450 (450K) BeadChip array. Using the GoDMC Atlas [46], we identified 446 clumped (per CpG), genome-wide significant ($p < 5e-8$) mQTLs and removed indels and duplicate mQTLs, retaining the mQTL within a duplicate set with the smallest p-value or largest absolute effect size for duplicate mQTLs with p=0, leaving 377 unique mQTLs. Finally, following matching for SNPs in our UK Biobank target dataset, removing amibiguous SNPs and further clumping ($r^2 = 0.1$; kb = 250), there were 189 SNPs in females and 188 SNPs in males.

BASE

SNP rsID	CpG ID	A1	A2	Beta(A1)	Direction (Famine DMRs)	Beta(A1) Direction
rs1	cg100	T	C	1.50	+1	1.50
rs2	cg200	A	T	2.00	-1	-2.00
rs3	cg300	G	C	-1.20	+1	-1.20
rs4	cg400	C	T	-3.00	-1	3.00

rs1: A1 -> higher methylation in mQTL atlas (+) and famine group (+)
rs2: A1 -> higher methylation in mQTL atlas (+) and lower in famine group (-)
rs3: A1 -> lower methylation in mQTL atlas (-) and higher in famine group (+)
rs4: A1 -> lower methylation in mQTL atlas (-) and lower in famine group (-)

TARGET

SUBID	rs1	rs2	rs3	rs4	Score
A	TT (1.5 * 2) = 3	AT (-2 * 1) = -2	GC (-1.2 * 1) = -1.2	CT (3 * 1) = 3	2.8
B	TC (1.5 * 1) = 1.5	AA (-2 * 2) = -4	GG (-1.2 * 2) = -2.4	CC (3 * 2) = 6	1.1
C	CC (1.5 * 0) = 0	AT (-2 * 1) = -2	CC (-1.2 * 0) = 0	TT (3 * 0) = 0	-2
D	TC (1.5 * 1) = 1.5	TT (-2 * 0) = 0	GG (-1.2 * 2) = -2.4	TT (3 * 0) = 0	-0.9

Fig. S7B. Constructing “famine” polygenic scores. In the toy examples illustrated here, we have 4 hypothetical mQTLs in our base dataset and 4 hypothetical subjects in our target dataset. The effect sizes (Beta[A1]) are to be obtained from the GoDMC mQTL atlas and these are weighted by the directionality of the effects from the DMRs reported by [28] (+1: hypermethylation among famine-exposed, relative to controls; -1: hypomethylation among famine-exposed, relative to controls). The famine scores are computed for each participant by summing up effect alleles per SNP, multiplied by the respective famine-weighted effect sizes.

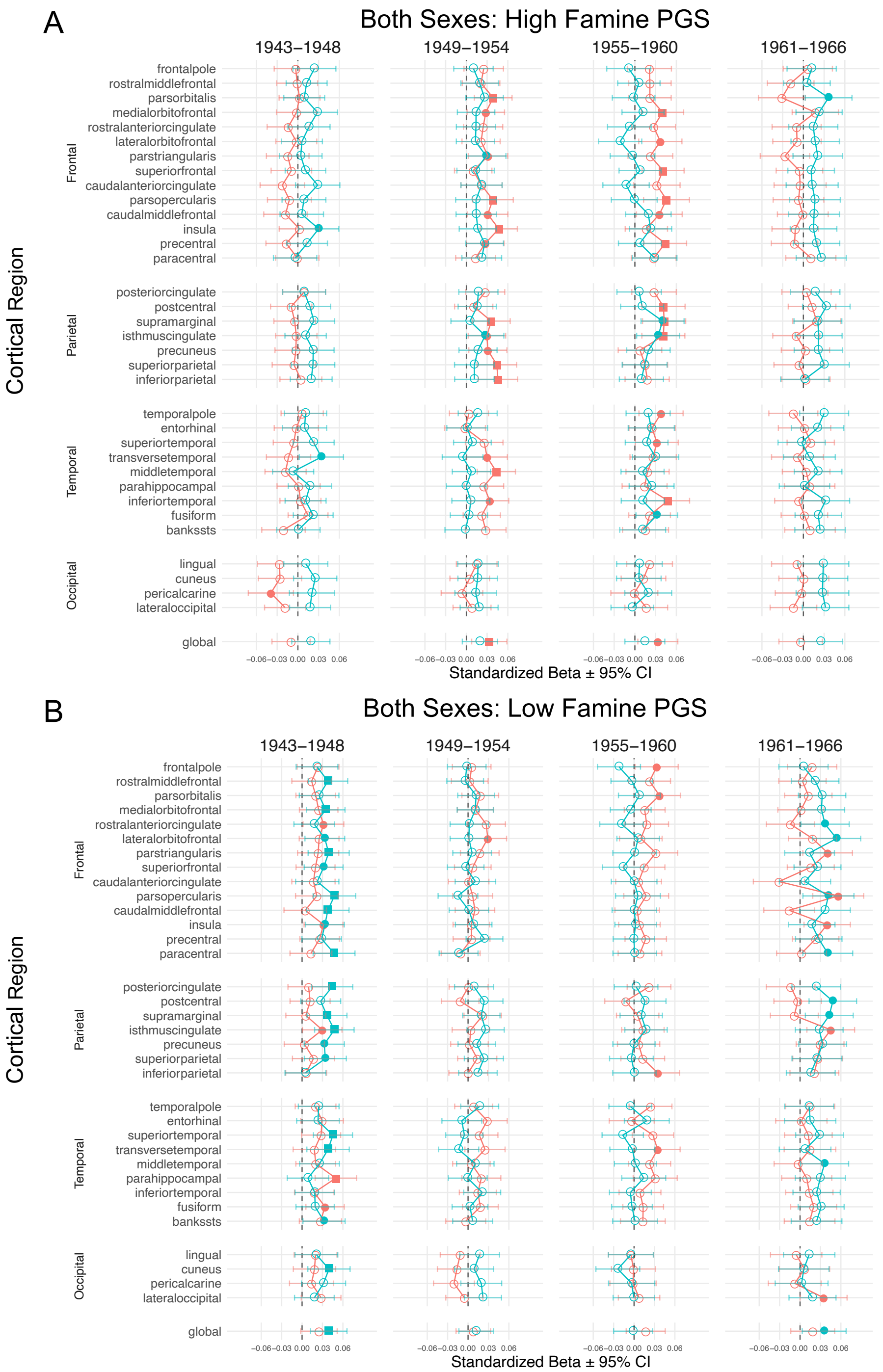

Fig. S8. Famine-mQTL PGS median split stratified associations between PGSs for fetal (red) and maternal (cyan) effects on birthweight and phenotypic cortical SA in the whole sample, segmented by year of birth period. The analyses were conducted in the following birth year periods: 1943-1948 (high famine: n = 3424, low famine: n = 3464), 1949-1954 (high: n = 4001, low: n = 4047), 1955-1960 (high: n = 3179, low: n = 3170), and 1961-1966 (high: n = 2503, low: n = 2468), with the high famine-mQTL results in the top panel (A) and the low famine-mQTL results in the bottom panel (B). The significance levels are indicated by filled squares ($p_{FDR} < 0.05$), filled circles ($p < 0.05$), and open circles ($p > 0.05$).

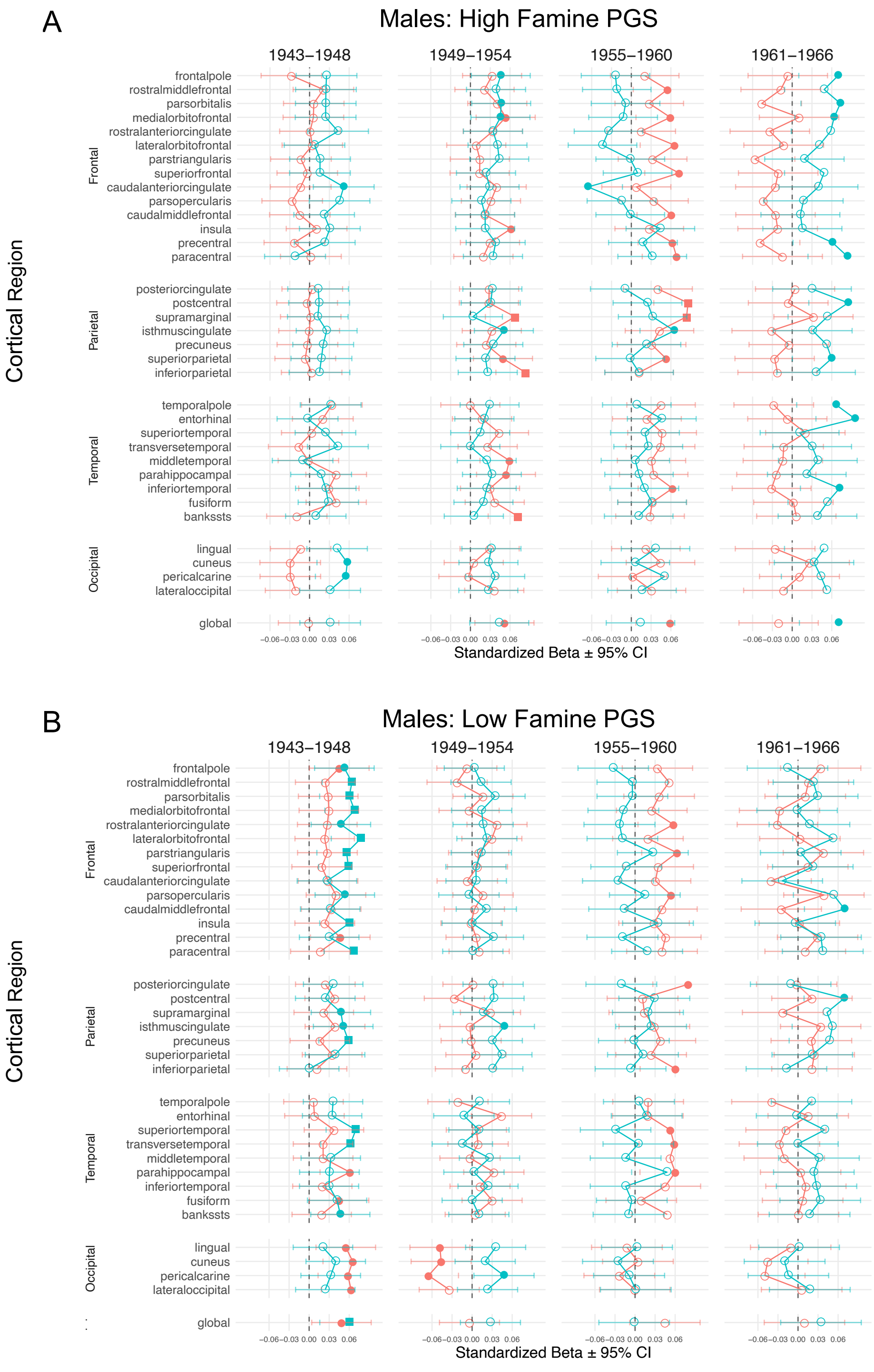

Fig. S9. Famine-mQTL PGS median split stratified associations between PGSs for fetal (red) and maternal (cyan) effects on birthweight and phenotypic cortical SA in males, segmented by year of birth period. The analyses were conducted in the following birth year periods: 1943-1948 (high: n = 1830, low: n = 1941), 1949-1954 (high: n = 1930, low: n = 1907), 1955-1960 (high: n = 1435, low: n = 1386), and 1961-1966 (high: n = 1078, low: n = 1076), with the high famine-mQTL results in the top panel (A) and the low famine-mQTL results in the bottom panel (B). The significance levels are indicated by filled squares ($p_{FDR} < 0.05$), filled circles ($p < 0.05$), and open circles ($p > 0.05$).

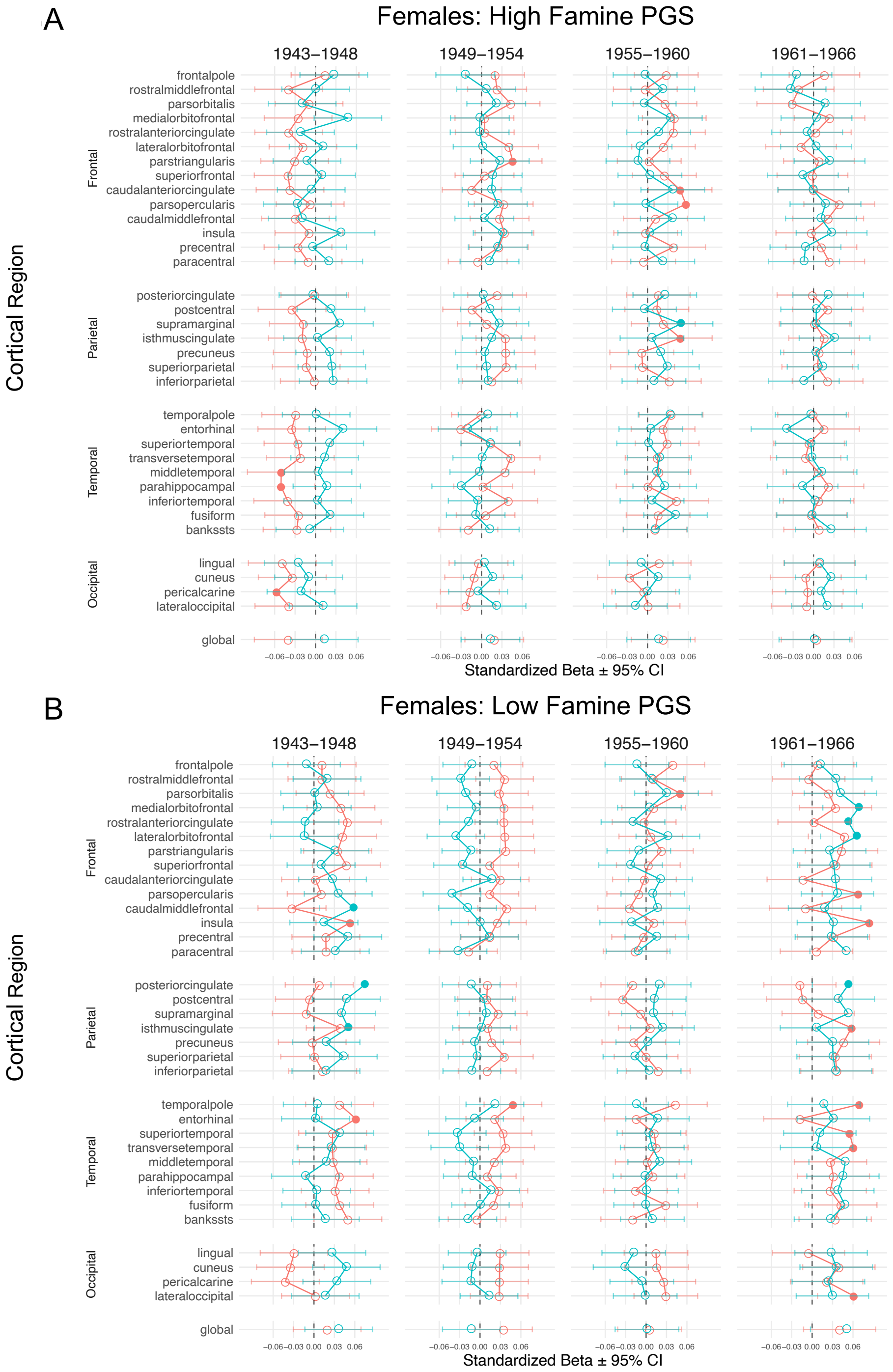

Fig. S10. Famine-mQTL PGS median split stratified associations between PGSs for fetal (red) and maternal (cyan) effects on birthweight and phenotypic cortical SA in females, segmented by year of birth period. The analyses were conducted in the following birth year periods: 1943-1948 (high: n = 1565, low: n = 1552), 1949-1954 (high: n = 2066, low: n = 2145), 1955-1960 (high: n = 1761, low: n = 1767), and 1961-1966 (high: n = 1443, low: n = 1374), with the high famine-mQTL results in the top panel (A) and the low famine-mQTL results in the bottom panel (B). The significance levels are indicated by filled squares ($p_{FDR} < 0.05$), filled circles ($p < 0.05$), and open circles ($p > 0.05$).

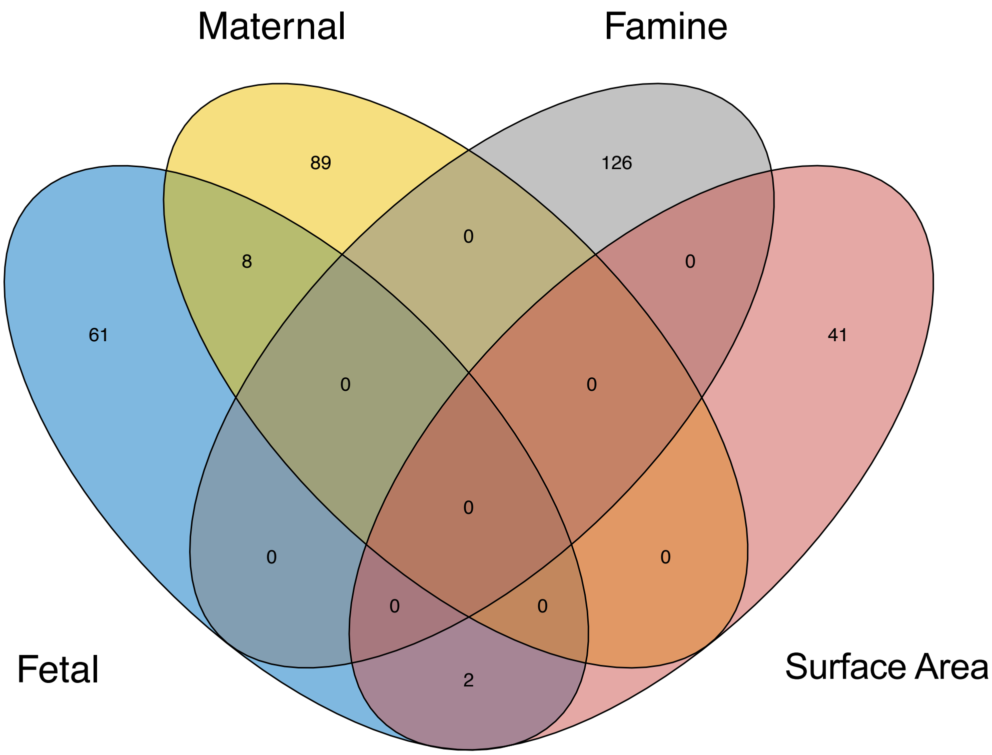

Fig. S11. Venn diagram of gene lists. There were 98 maternal, 71 fetal, 128 famine, and 40 surface area genes, mapped from SNPs using FUMA-GWAS.

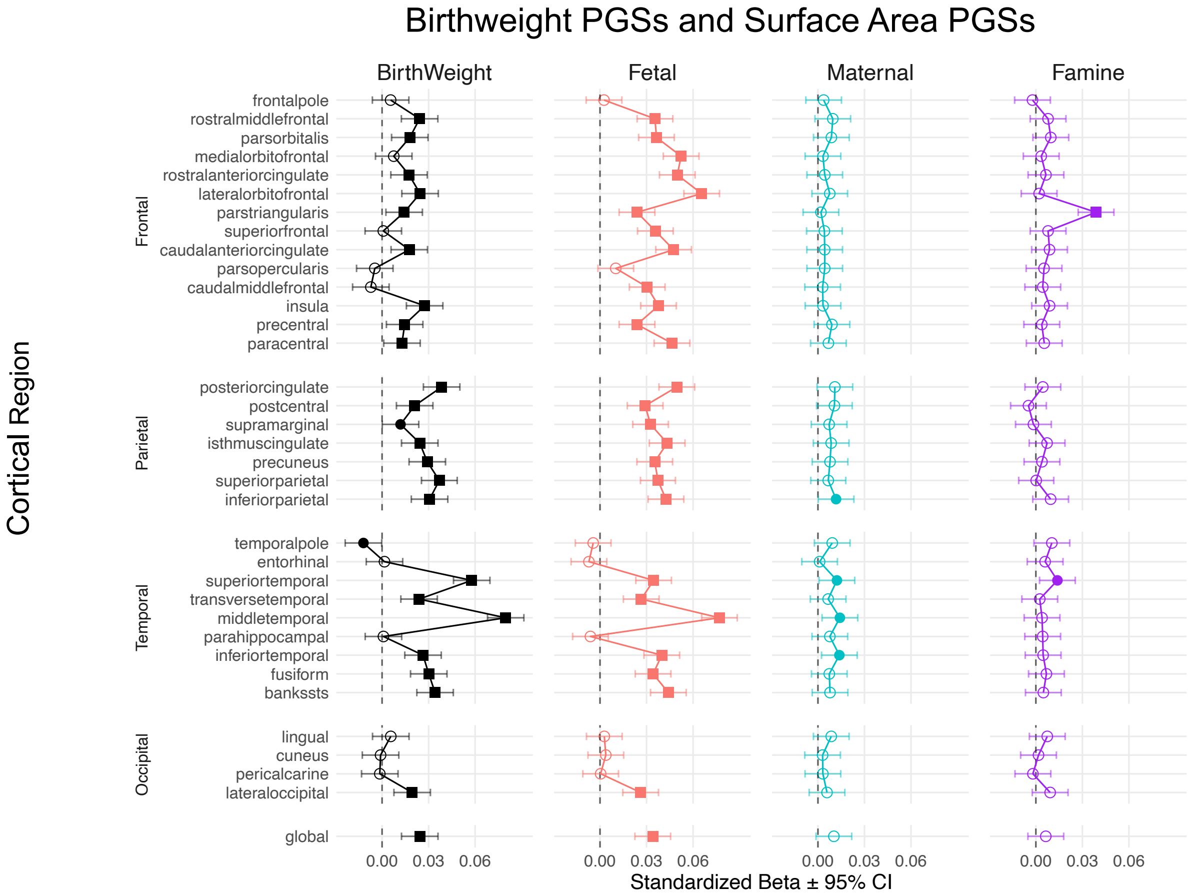

Fig. S12. Associations between PGSs for birthweight (black), fetal (red), maternal (cyan), and famine (purple) and polygenic cortical SA (total n = 29,047). The p-value threshold for SNP inclusion was $p < 5e\text{-}8$ for all the PGSs. The significance levels are indicated by filled squares ($p_{FDR} < 0.05$), filled circles ($p < 0.05$), and open circles ($p > 0.05$).

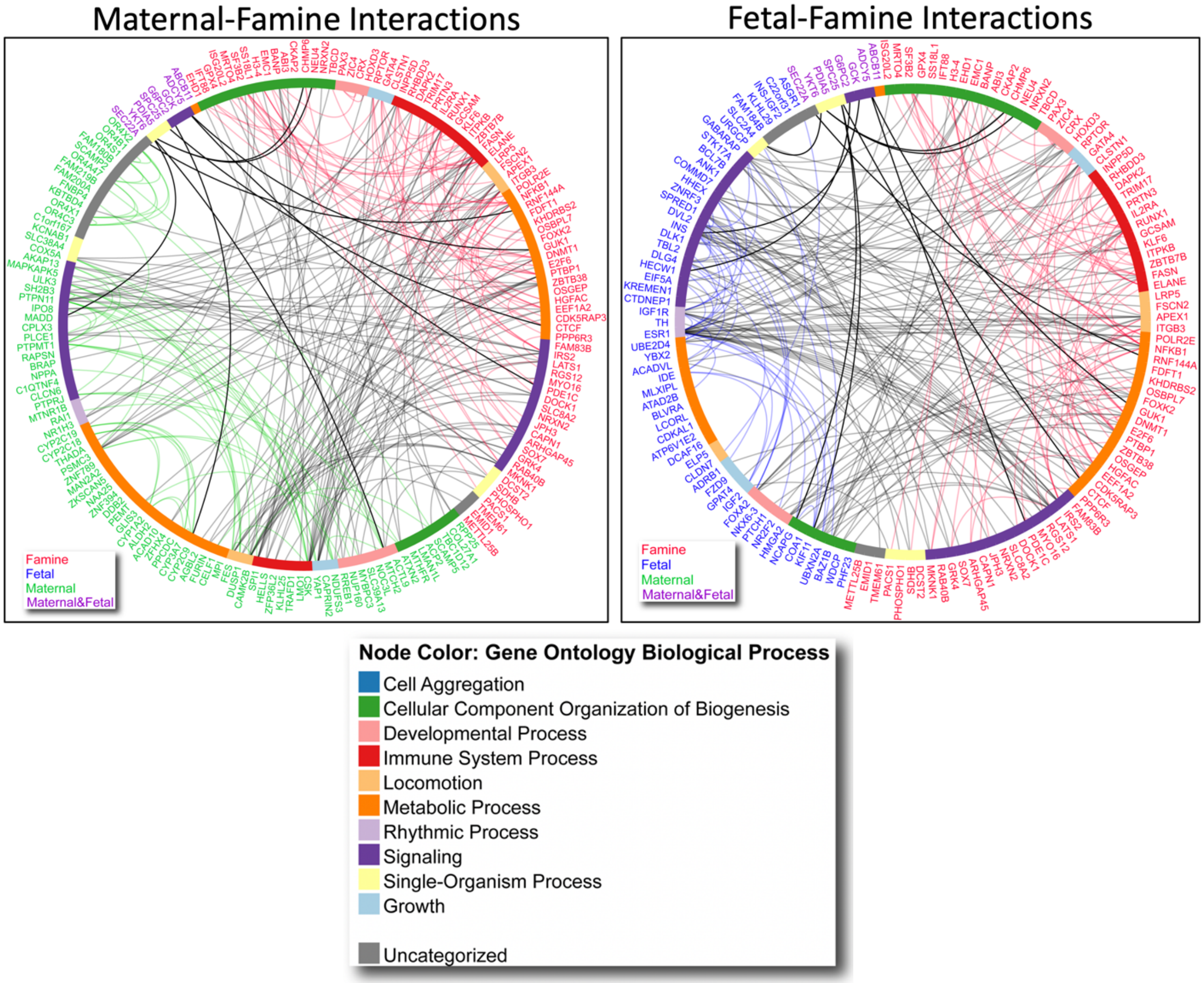

Fig. S13. Protein-protein interactions among maternal-famine DEGs (left) and fetal-famine DEGs (right). The protein names and within-network interaction edges are color coded as follows: famine (red), maternal (green), and fetal (blue). The purple protein names indicate those shared between maternal and fetal, and black edges indicate interactions with these proteins. All other between-network interactions are represented with grey edges. For clarity, self-interactions are not presented. The color of nodes indicates the Gene Ontology Biological Process. Protein-protein interactions were retrieved from the Integrated Interactions Database and the networks were visualized using NAViGaTOR, and finalized in Adobe Illustrator with legends.

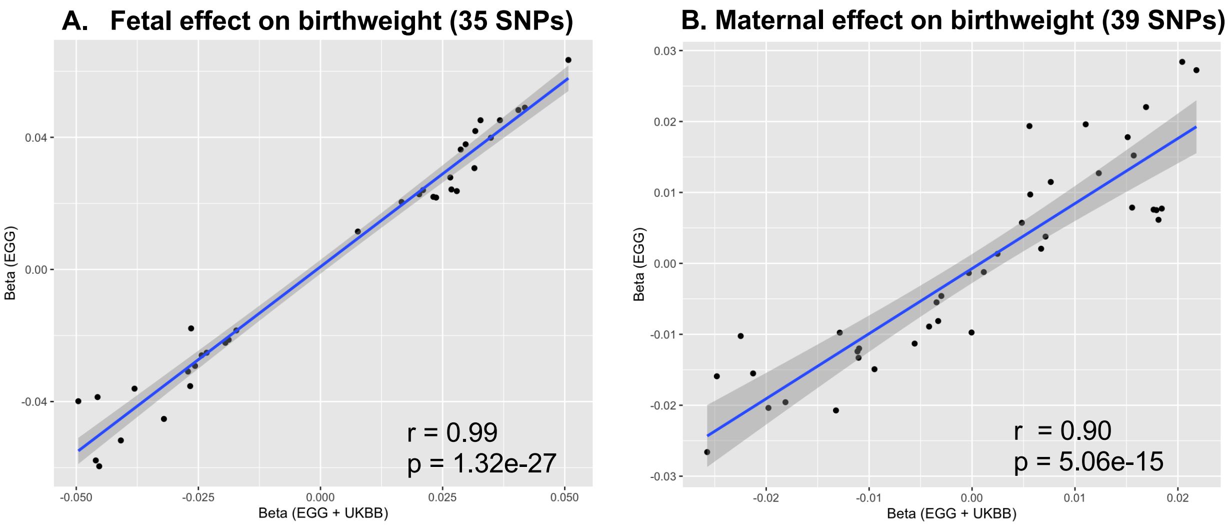

Fig. S14. Correlations between SNP effect sizes between sample with and without UK Biobank participants. First, we obtained summary GWAS statistics for birthweight based on the EGG Consortium alone, excluding the UK Biobank, from Warrington et al. (2019). Summary GWAS statistics for the maternal and fetal effects, excluding the UK Biobank cohort, were not available. Thus, using the birthweight GWAS of the EGG cohort alone, we filtered for the 35 “fetal effect” and 39 “maternal effect” SNPs, and identified that these were highly correlated between the original cohort (EGG and UK Biobank) and EGG cohort alone (fetal: $r = 0.99$, $p = 1.32e-27$; maternal: $r = 0.90$, $p = 5.06e-15$).

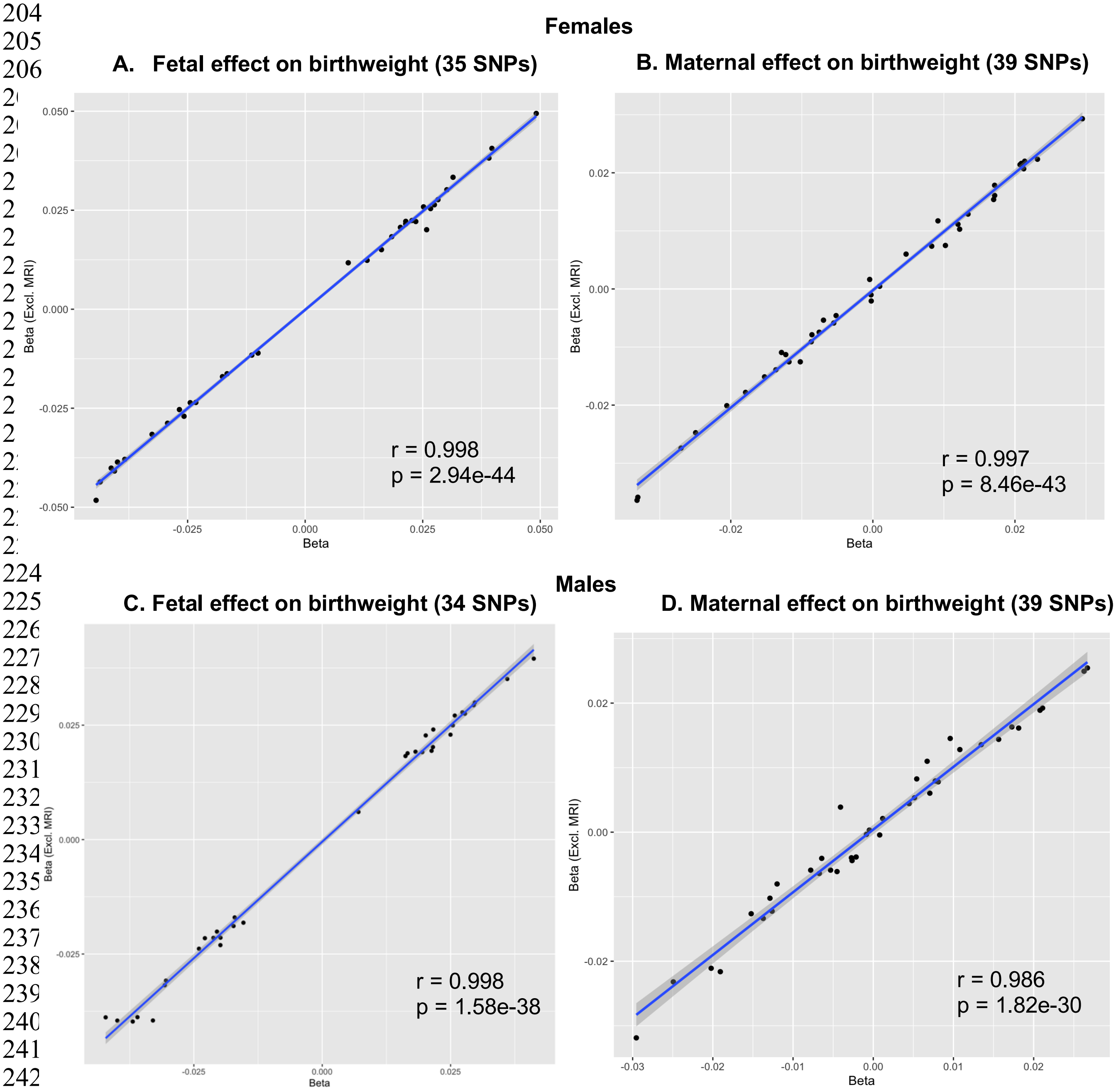

Fig. S15. Correlations between SNP effect sizes between total UKBB sample and sample excluding the participants with MRI data. We conducted sex-specific GWASs of birthweight for the total sample in the UK Biobank and for the sample excluding those with MRI data, and assessed the correlations of the effect sizes of the SNPs between the two samples. As indicated in the following plots, the effect sizes between the two samples were very highly correlated (all $r \geq 0.99$) for both the fetal and maternal SNPs.
