# Supplementary material for: Intrauterine growth and the tangential expansion of the human cerebral cortex in times of food scarcity and abundance
Source: Nat Commun. 2024 Feb 13;15:1205. doi: 10.1038/s41467-024-45409-6 (PMC10864407; doi:10.1038/s41467-024-45409-6)
Supplement: Supplementary file 3 — Description of Additional Supplementary Files [file 41467_2024_45409_MOESM3_ESM.pdf]

Description of Additional Supplementary Files

File Name: Supplementary Data 1
Description: The top pathways identified as enriched from the Gene Ontology (GO) analysis of within-network (direct) protein- protein interactions of fetal (green), maternal (yellow), and famine (orange) proteins. The results are provided for (A) biological processes, (B) cellular component, and (C) molecular function.

File Name: Supplementary Data 2
Description: The complete list of protein-protein interactions, including all interaction sources, for the (A) fetal, (B) maternal and (C) famine genes.

File Name: Supplementary Software 1
Description: The custom R code for selecting the mQTLs for the famine PGS (see also the *famine polygenic score* section in the *Methods*).
