# Supplementary material for: Intrauterine growth and the tangential expansion of the human cerebral cortex in times of food scarcity and abundance
Source: Nat Commun. 2024 Feb 13;15:1205. doi: 10.1038/s41467-024-45409-6 (PMC10864407; doi:10.1038/s41467-024-45409-6)
Supplement: Supplementary file 6 — Reporting Summary [file 41467_2024_45409_MOESM6_ESM.pdf]

nature portfolio

Corresponding author(s): Tomas Paus

Last updated by author(s): Jan 5, 2024

Reporting Summary

Nature Portfolio wishes to improve the reproducibility of the work that we publish. This form provides structure for consistency and transparency in reporting. For further information on Nature Portfolio policies, see our Editorial Policies and the Editorial Policy Checklist.

Statistics

For all statistical analyses, confirm that the following items are present in the figure legend, table legend, main text, or Methods section.

n/a	Confirmed	
☐	☒	The exact sample size (*n*) for each experimental group/condition, given as a discrete number and unit of measurement
☒	☐	A statement on whether measurements were taken from distinct samples or whether the same sample was measured repeatedly
☐	☒	The statistical test(s) used AND whether they are one- or two-sided<br>*Only common tests should be described solely by name; describe more complex techniques in the Methods section.*
☐	☒	A description of all covariates tested
☐	☒	A description of any assumptions or corrections, such as tests of normality and adjustment for multiple comparisons
☐	☒	A full description of the statistical parameters including central tendency (e.g. means) or other basic estimates (e.g. regression coefficient) AND variation (e.g. standard deviation) or associated estimates of uncertainty (e.g. confidence intervals)
☐	☒	For null hypothesis testing, the test statistic (e.g. *F*, *t*, *r*) with confidence intervals, effect sizes, degrees of freedom and *P* value noted<br>*Give P values as exact values whenever suitable.*
☒	☐	For Bayesian analysis, information on the choice of priors and Markov chain Monte Carlo settings
☒	☐	For hierarchical and complex designs, identification of the appropriate level for tests and full reporting of outcomes
☐	☒	Estimates of effect sizes (e.g. Cohen's *d*, Pearson's *r*), indicating how they were calculated

Our web collection on statistics for biologists contains articles on many of the points above.

Software and code

Policy information about availability of computer code

Data collection	Integrated Interactions Database (IID) version 2021-05 (http://opthid.utoronto.ca/iid)
Data analysis	We used PRSice-2, FreeSurfer, R version 4.1.1, the R packages 'tidyverse' version 1.3.2. and 'ukbtools' version 0.11.3, NAViGaTOR 48 ver. 3.0.17, and Adobe Illustrator ver. 27.2.

For manuscripts utilizing custom algorithms or software that are central to the research but not yet described in published literature, software must be made available to editors and reviewers. We strongly encourage code deposition in a community repository (e.g. GitHub). See the Nature Portfolio guidelines for submitting code & software for further information.

Data

Policy information about availability of data

All manuscripts must include a data availability statement. This statement should provide the following information, where applicable:

- Accession codes, unique identifiers, or web links for publicly available datasets
- A description of any restrictions on data availability
- For clinical datasets or third party data, please ensure that the statement adheres to our policy

The data can be provided by the UK Biobank pending scientific review and a completed material transfer agreement. Applications for access to the data can be completed at: https://www.ukbiobank.ac.uk/enable-your-research/apply-for-access. Physical protein-protein interactions were obtained from the Integrated Interactions Database (IID) version 2021-05 (http://opthid.utoronto.ca/iid).

Research involving human participants, their data, or biological material

Policy information about studies with human participants or human data. See also policy information about sex, gender (identity/presentation), and sexual orientation and race, ethnicity and racism.

Reporting on sex and gender	All of our analyses were conducted in each sex as well as the overall sample, with sex as a covariate. Participants with a mismatch between genetic and reported sex (n = 197) or sex chromosomal aneuploidy (n = 651) were excluded. Overall, the study included 14,905 females and 14,142 males who passed inclusion criteria, genetic quality control, and who had values for cortical surface area.
Reporting on race, ethnicity, or other socially relevant groupings	To minimize the confound of population stratification in genetic analyses, the study was restricted to participants of European descent.
Population characteristics	The UK Biobank is a large richly phenotyped and genotyped cohort of ~500k individuals, aged 37-73. There were 14,905 females and 14,142 males with genetic and MRI data of European ancestry, following exclusions (described below).
Recruitment	Participants were recruited from 22 assessment centers across Scotland, England, and Wales, beginning with invitation letters to those aged 40-69, for the baseline assessment. Moreover, participants were invited for follow-ups, including 50k participants who have underwent imaging. It has been reported that UK Biobank participants are healthier and have higher educational attainment than the general population in the UK (Schoeler et al., Nat Hum Behav, 2023).
Ethics oversight	The UK Biobank study was approved by the North West Multi-centre Research Ethics Committee as a Research Tissue Bank (see: https://www.ukbiobank.ac.uk/learn-more-about-uk-biobank/about-us/ethics). This study was approved under the UK Biobank Resource Application Number 43688 and by local ethics committees at the Research Institute of the Hospital for Sick Children (SickKids) and the Centre Hospitalier Universitaire (CHU) Sainte-Justine.

Note that full information on the approval of the study protocol must also be provided in the manuscript.

Field-specific reporting

Please select the one below that is the best fit for your research. If you are not sure, read the appropriate sections before making your selection.

☒ Life sciences ☐ Behavioural & social sciences ☐ Ecological, evolutionary & environmental sciences

For a reference copy of the document with all sections, see nature.com/documents/nr-reporting-summary-flat.pdf

Life sciences study design

All studies must disclose on these points even when the disclosure is negative.

Sample size	The UK Biobank comprises approximately half a million participants. Restricting to those with magnetic resonance imaging (MRI) data, passing genetic quality control, and meeting inclusion criteria, there were 14,905 females and 14,142 males. This is the largest sample size available to us given our criteria.
Data exclusions	Participants without genetic information or available genetic sex, heterozygosity or missingness outliers, a mismatch between genetic and reported sex, sex chromosomal aneuploidy, non-European ancestry were excluded. Moreover, participants with close kinship, born outside the UK, weighing less than 2,500 g at birth, or as part of multiple births, were excluded.
Replication	Given the characteristics of the UK Biobank sample (birth years overlapping with WWII, very large sample size, genetic and MRI data), a similar cohort for replication is not easily obtained, but this should be addressed by future studies.
Randomization	This is not relevant as it is not a controlled experiment.
Blinding	This is not relevant as it is not a controlled experiment.

Reporting for specific materials, systems and methods

We require information from authors about some types of materials, experimental systems and methods used in many studies. Here, indicate whether each material, system or method listed is relevant to your study. If you are not sure if a list item applies to your research, read the appropriate section before selecting a response.

Materials & experimental systems

n/a	Involved in the study
☒	☐ Antibodies
☒	☐ Eukaryotic cell lines
☒	☐ Palaeontology and archaeology
☒	☐ Animals and other organisms
☒	☐ Clinical data
☒	☐ Dual use research of concern
☒	☐ Plants

Methods

n/a	Involved in the study
☒	☐ ChIP-seq
☒	☐ Flow cytometry
☐	☒ MRI-based neuroimaging

Magnetic resonance imaging

Experimental design

Design type: N/A (structural MRI)

Design specifications: N/A (structural MRI)

Behavioral performance measures: N/A (structural MRI)

Acquisition

Imaging type(s): Structural

Field strength: 3 Tesla

Sequence & imaging parameters: 1x1x1 mm resolution and 208x256x256 field-of-view. 3D MPRAGE, sagittal, in-plane acceleration iPAT=2, prescan-normalise.

Area of acquisition: Whole brain

Diffusion MRI: ☐ Used ☒ Not used

Preprocessing

Preprocessing software: FreeSurfer 6.0.1.

Normalization: Non-linear normalization

Normalization template: ICBM152

Noise and artifact removal: Motion-correction

Volume censoring: FreeSurfer 6.0.1.

Statistical modeling & inference

Model type and settings: Multiple regression with surface area as the outcome, polygenic score as the predictor, and principle components of genetic ancestry, age, and sex as covariates

Effect(s) tested: N/A (structural)

Specify type of analysis: ☐ Whole brain ☐ ROI-based ☒ Both

Anatomical location(s): Desikan-Killiany atlas

Statistic type for inference: Analyzed 34 regions + total cortical surface area, and applied an FDR correction

(See Eklund et al. 2016)

Correction: FDR

Models & analysis

n/a	Involved in the study
☒	☐ Functional and/or effective connectivity
☒	☐ Graph analysis
☒	☐ Multivariate modeling or predictive analysis
